# Heel reconstruction with free instep flap: a case report

**DOI:** 10.1186/1752-1947-8-319

**Published:** 2014-09-26

**Authors:** Lifeng Liu, Yu Zhou, Xuexin Cao, Xuecheng Cao, Jinfang Cai

**Affiliations:** 1 Orthopedic Department, General Hospital of Jinan Military Command, No.25, Shifan Road, Jinan 250031, China; 2 Clinical Medicine Department, Liaoning Medical College, No.40, Songpo Road (3rd), Jinzhou 121001, China

**Keywords:** Free instep flap, Heel reconstruction, Medial plantar artery, Trauma

## Abstract

**Introduction:**

Reconstruction of weight-bearing heel defects remains a challenge because of the unique characteristics of the plantar skin. Though numerous surgical reconstructive options have been reported, the instep flap represents an ideal option and seems to be more acceptable to patients than others. However, when the heel defect expands to the instep area, the ipsilateral instep is not available for flap elevation. A free instep flap harvested from the contralateral foot can be a good solution, but this method has been scarcely reported.

**Case presentation:**

A 41-year-old Asian man presented to our institution with a soft-tissue lesion in the weight-bearing heel and instep area. His heel was reconstructed with a free instep flap from the other foot, end-to-side anastomosis of its medial plantar artery to the recipient posterior tibial artery and end-to-side coaptation of the cutaneous sensory fascicles of the flap to the medial plantar nerve.

**Conclusion:**

The flap survived successfully, and no ulceration occurred in the flap. At the last follow-up appointment at 30 months post-surgery, a very good functional and aesthetic outcome was verified, indicating that the suggested approach may prove to be the treatment of choice in selected cases of weight-bearing heel reconstruction.

## Introduction

Reconstruction of a weight-bearing heel is always a surgical challenge because of the anatomical characteristics and functional particularity of this region. The sole contains durable and thick glabrous skin, which makes it difficult to replace by unlike skin from distant sites. Numerous surgical reconstructive options have been described previously, such as skin graft, sural flap and instep flap. Each of them has its merits and demerits. Skin grafts cannot provide adequate and permanent coverage of a weight-bearing region and tend to breakdown. Sometimes the defects can easily be covered by an instep flap or a distally based sural neurocutaneous island flap [1,2]. However, the sural flap has its own disadvantages, including flap thickness, unlike tissues and limited protective sensation even after nerve anastomosis [3]. The instep flap was originally developed to cover moderate-size defects of the ipsilateral heel [4]. The most favorable feature of the instep flap is that it can provide ideal “like” tissue for heel reconstruction without compromising the weight-bearing area of the foot. However, in a situation where defects expand to the instep area, the ipsilateral instep is not available for flap elevation. Cross-foot instep island flaps [5] can be used in these cases, but they are associated with long-term immobilization and restrained positioning of the legs. It can be considered only as the final desperate possibility. In such cases, the free instep flap harvested from the other foot can be a good solution to cover the soft-tissue defects of the weight-bearing heel. In fact, only a limited number of cases of heel reconstruction with a free instep flap have been reported to date. In this case report, we present our experience with the use of a free instep flap for reconstruction of the weight-bearing heel region.

## Case presentation

**Figure 1:**
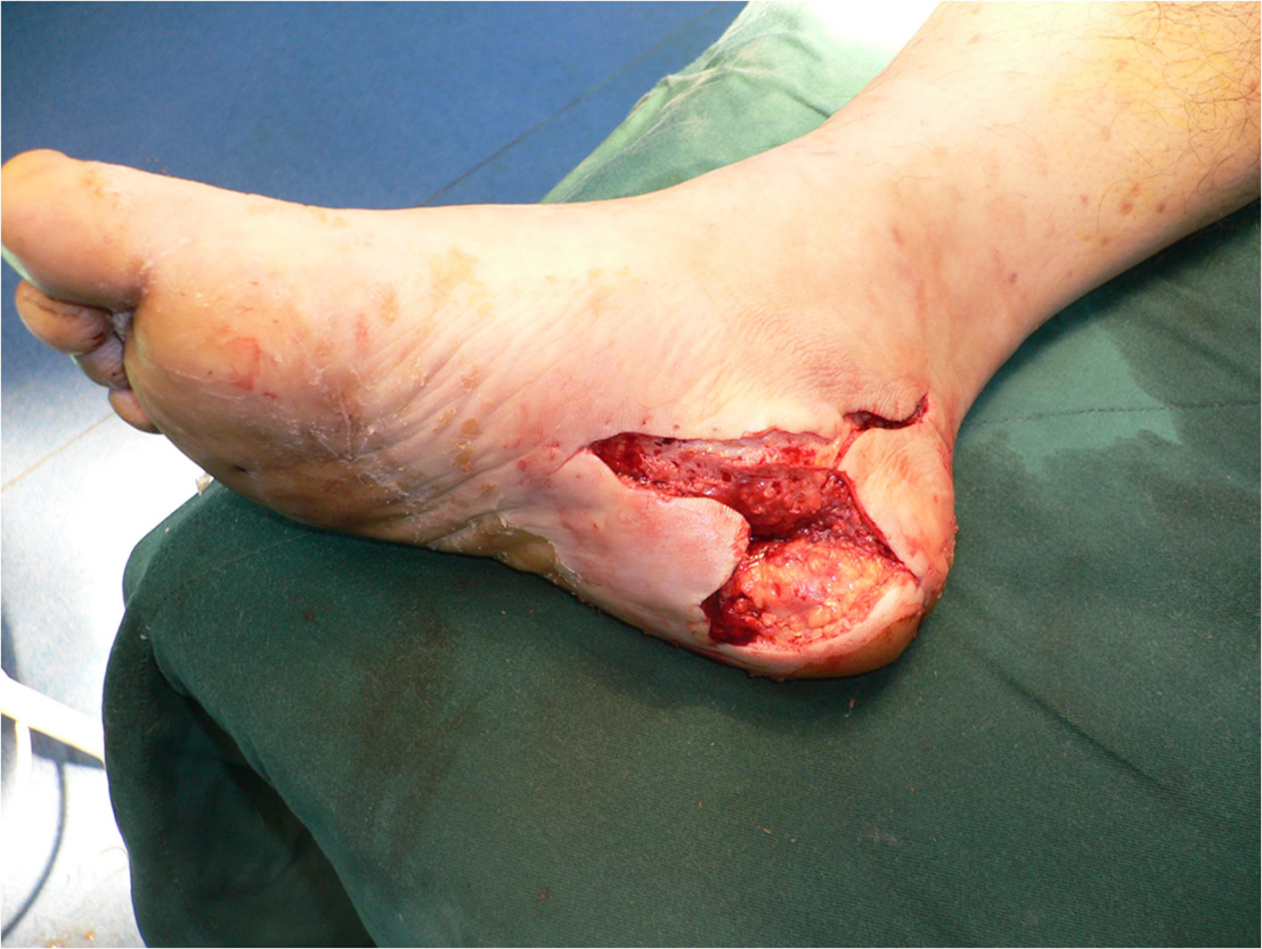
Pre-operative photograph showing the size of the soft-tissue defect (6cm×4cm).

**Figure 2:**
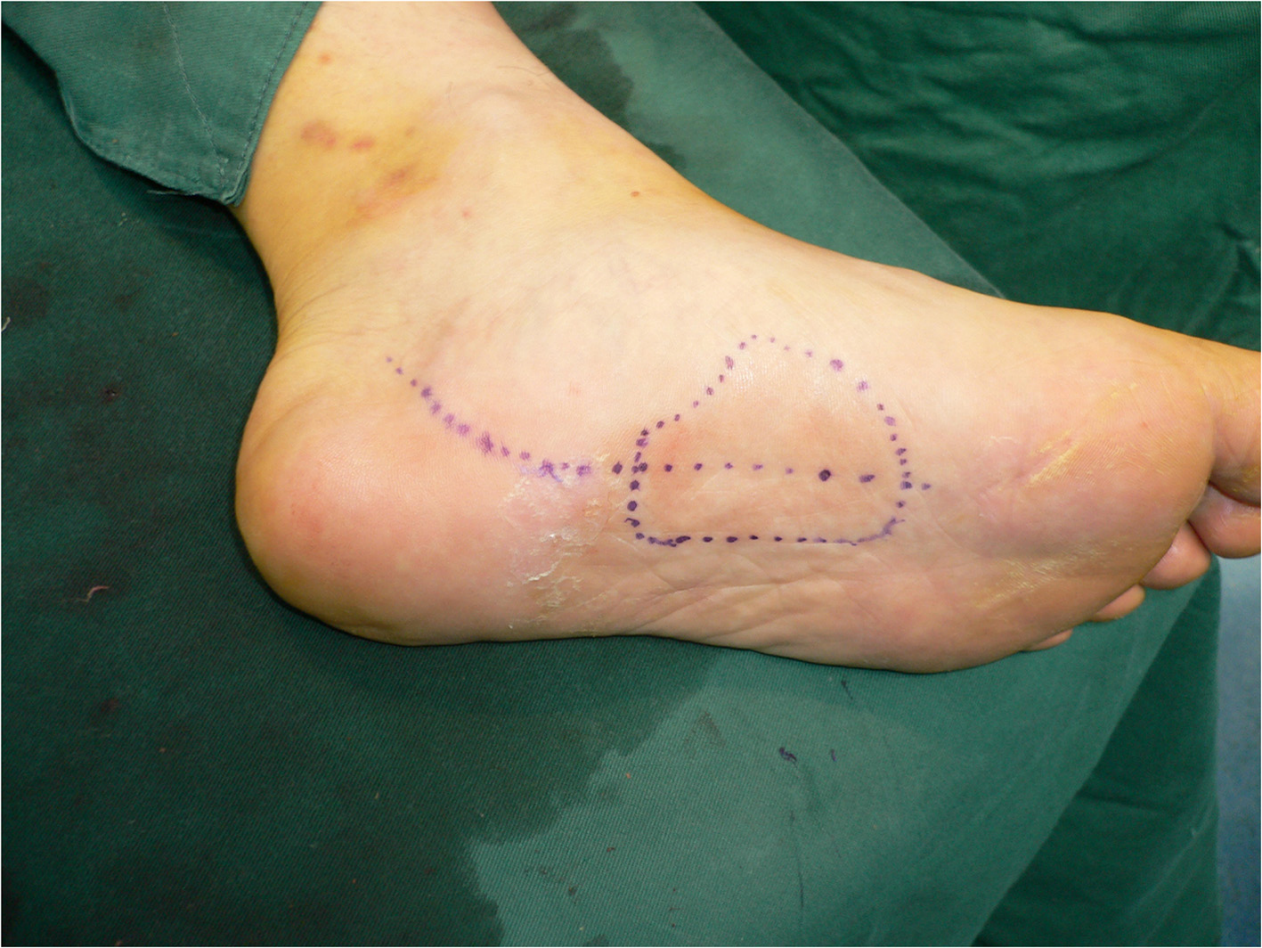
The instep flap (7cm×5cm) was designed according to a template made from the area of the defect.

**Figure 3:**
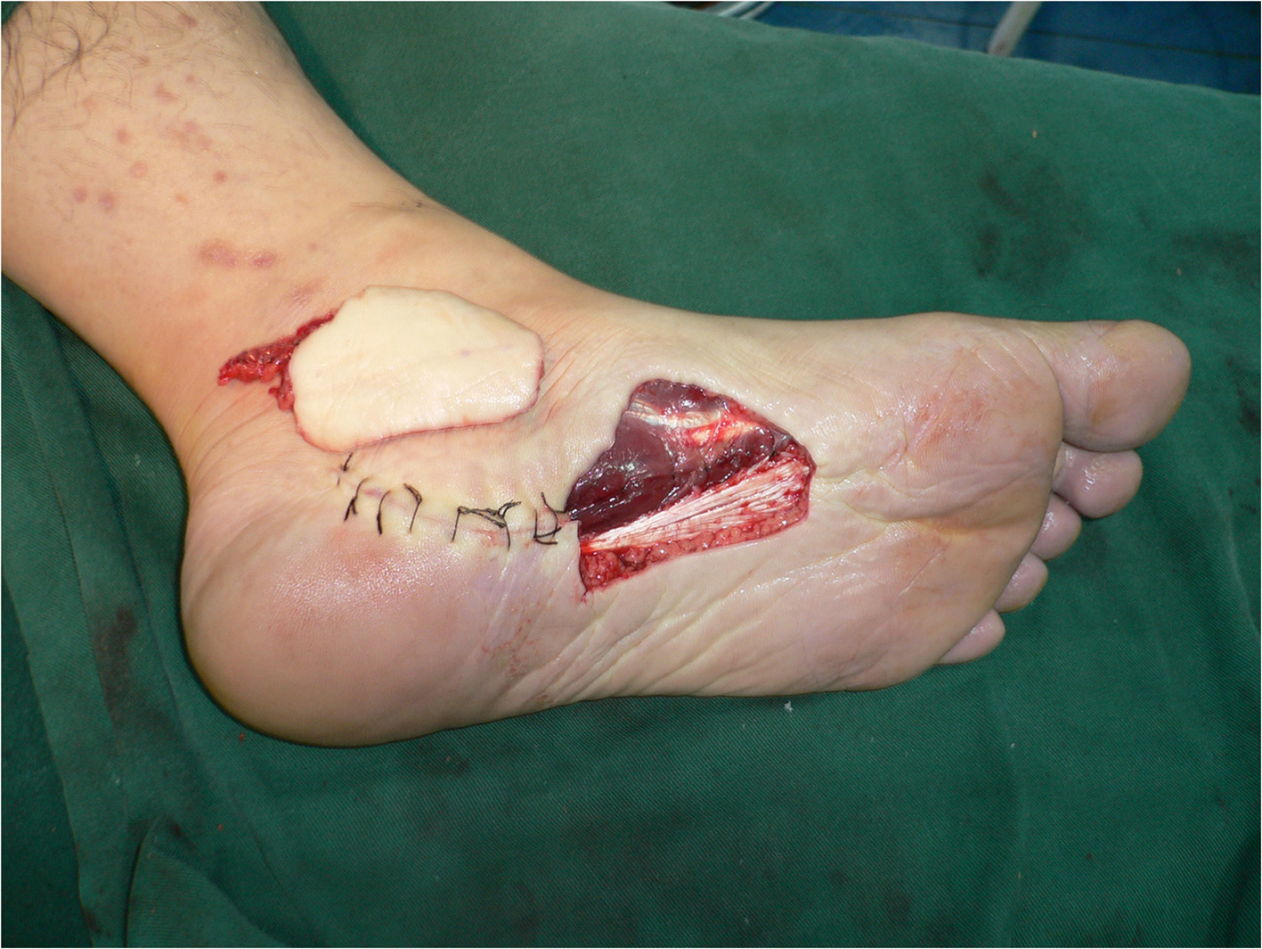
Intraoperative photograph taken after elevation of the instep flap.

**Figure 4:**
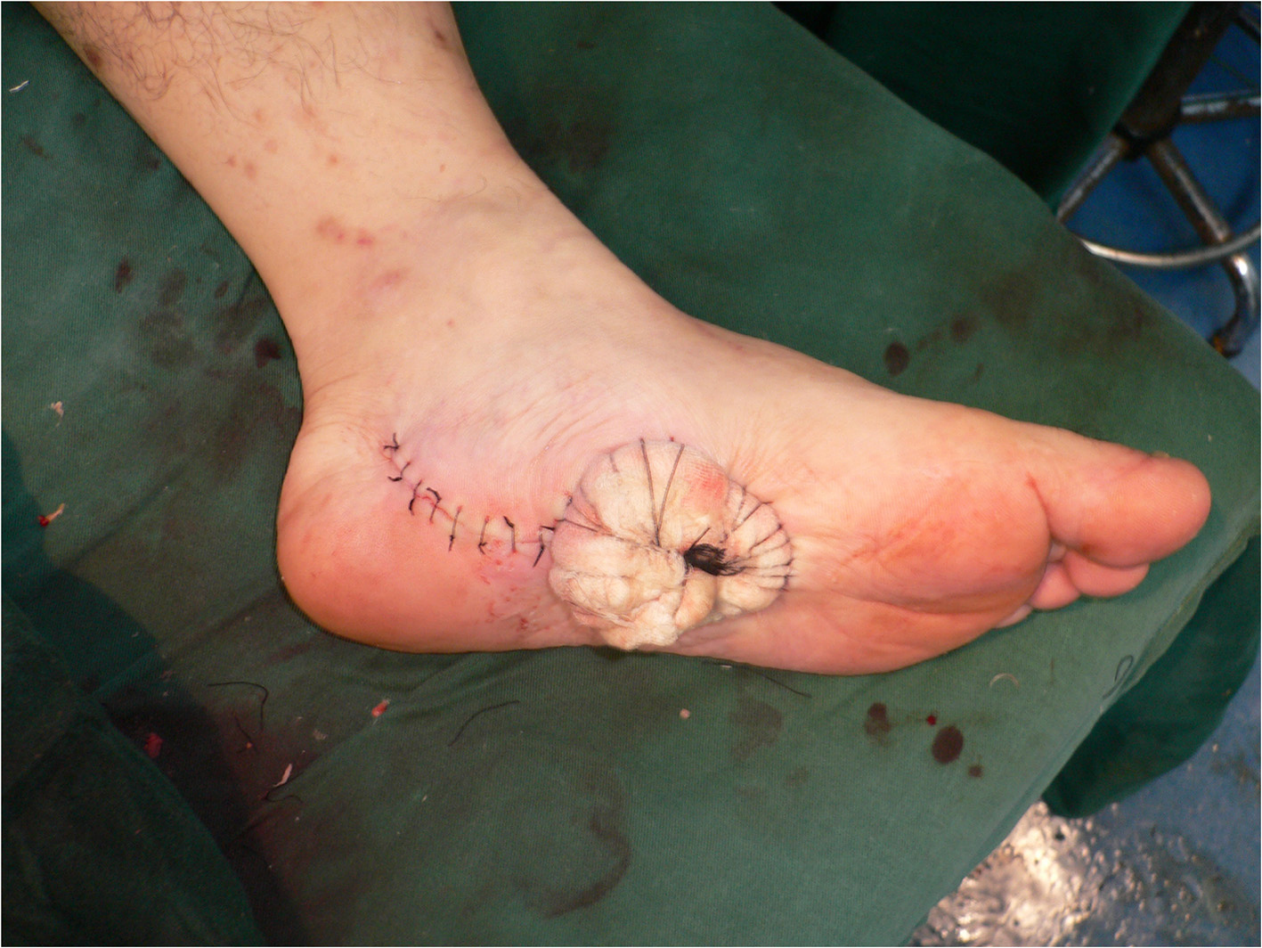
The donor site was covered with a split-thickness skin graft taken from the thigh.

A 41-year-old Asian man presented to our hospital after an automobile accident in which he sustained a severe crush injury to his right foot that resulted in formation of a soft-tissue lesion in the weight-bearing heel and instep area. After the debridement, the size of a full-thickness defect at the medial and anterior aspects of the heel measured 6cm in length and 4cm in width (Figure 1). Because the extensive zone of injury involved the proximal instep area, an ipsilateral instep island flap was not possible. Therefore, a plan was made to cover the defect using a free instep flap.The patient was placed in the prone position, and both legs were abducted for simultaneous flap elevation and preparation of the recipient site. The operation was performed under tourniquet control for better visualization. Debridement of the defect was performed, and the stumps of the posterior tibial artery and vein, as well as the tibial nerve, were indentified and dissected proximal to the defect. In harvesting the instep flap from the contralateral foot, the skin paddle was designed 7cm in length and 5cm in width and marked in the instep area (Figure 2), so that it was slightly larger than the defect to allow for flap-moulding. Flap dissection began with an incision along the distal border of the flap down to the fascia, which then was incised and dissected in a plane superficial to the abductor hallucis muscle. The distal branches of the medial plantar artery were carefully indentified and divided. The intermuscular septum between the abductor hallucis muscle and the flexor digitorum brevis containing the medial plantar artery, nerve and its fasciocutaneous perforators was raised in continuity with the flap. During flap dissection, the medial plantar nerve and the cutaneous branches of the first toe were preserved, but cutaneous nerve fascicles were retained within the flap. To obtain adequate length of these fascicles, we performed interfascicular dissection of the medial plantar nerve proximally. To facilitate flap harvest, the abductor hallucis muscle was divided at its origin, exposing the neurovascular pedicle deep into it. The neurovascular pedicle of the medial plantar artery and venae comitans was dissected proximally up to the common origin of the medial and lateral plantar artery from the posterior tibial artery (Figure 3). The abductor hallucis muscle was reattached, and the donor site was covered with a split-thickness skin graft taken from the thigh (Figure 4).

**Figure 5:**
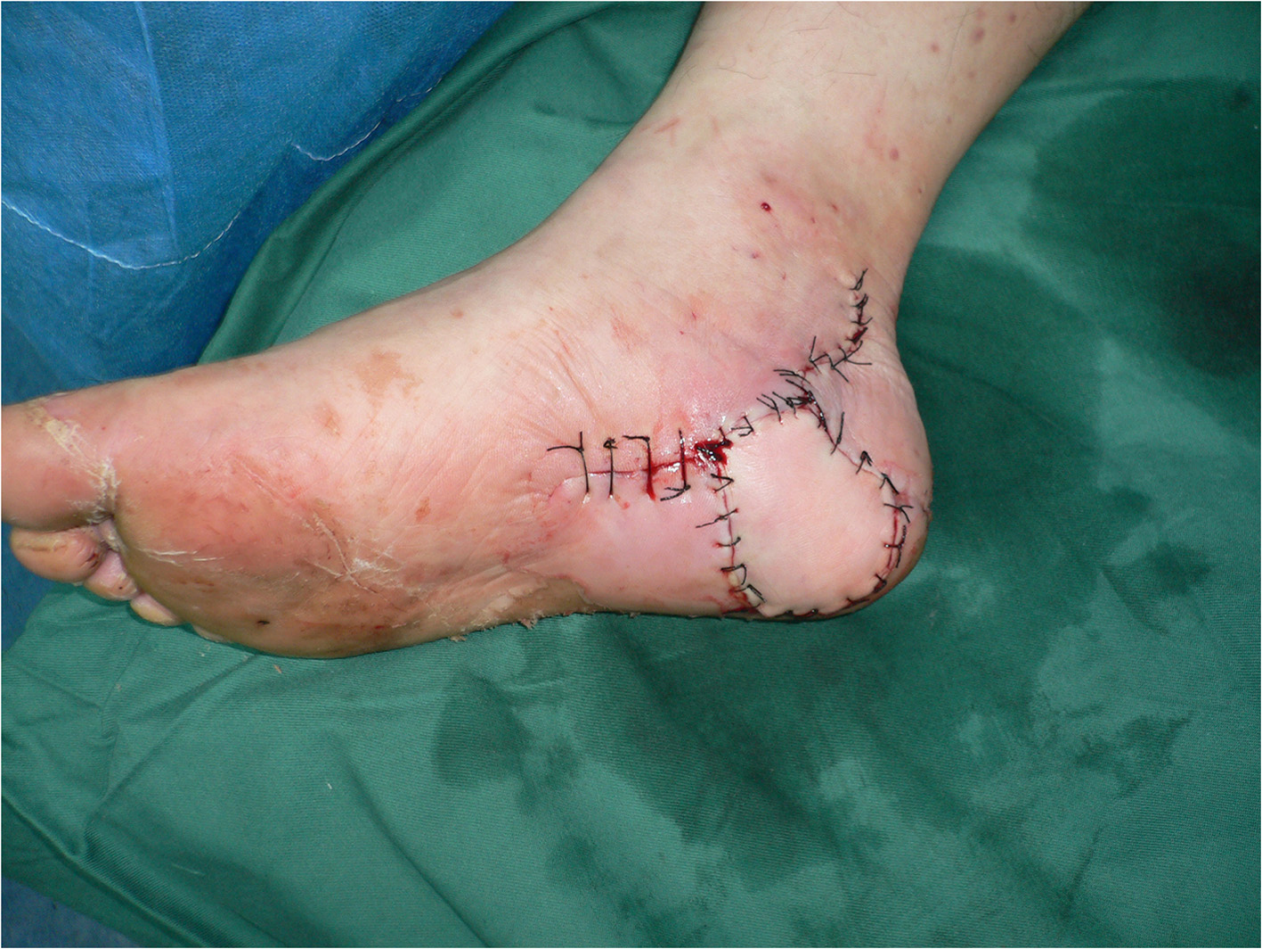
Post-operative photograph of coverage of the heel defect.

**Figure 6:**
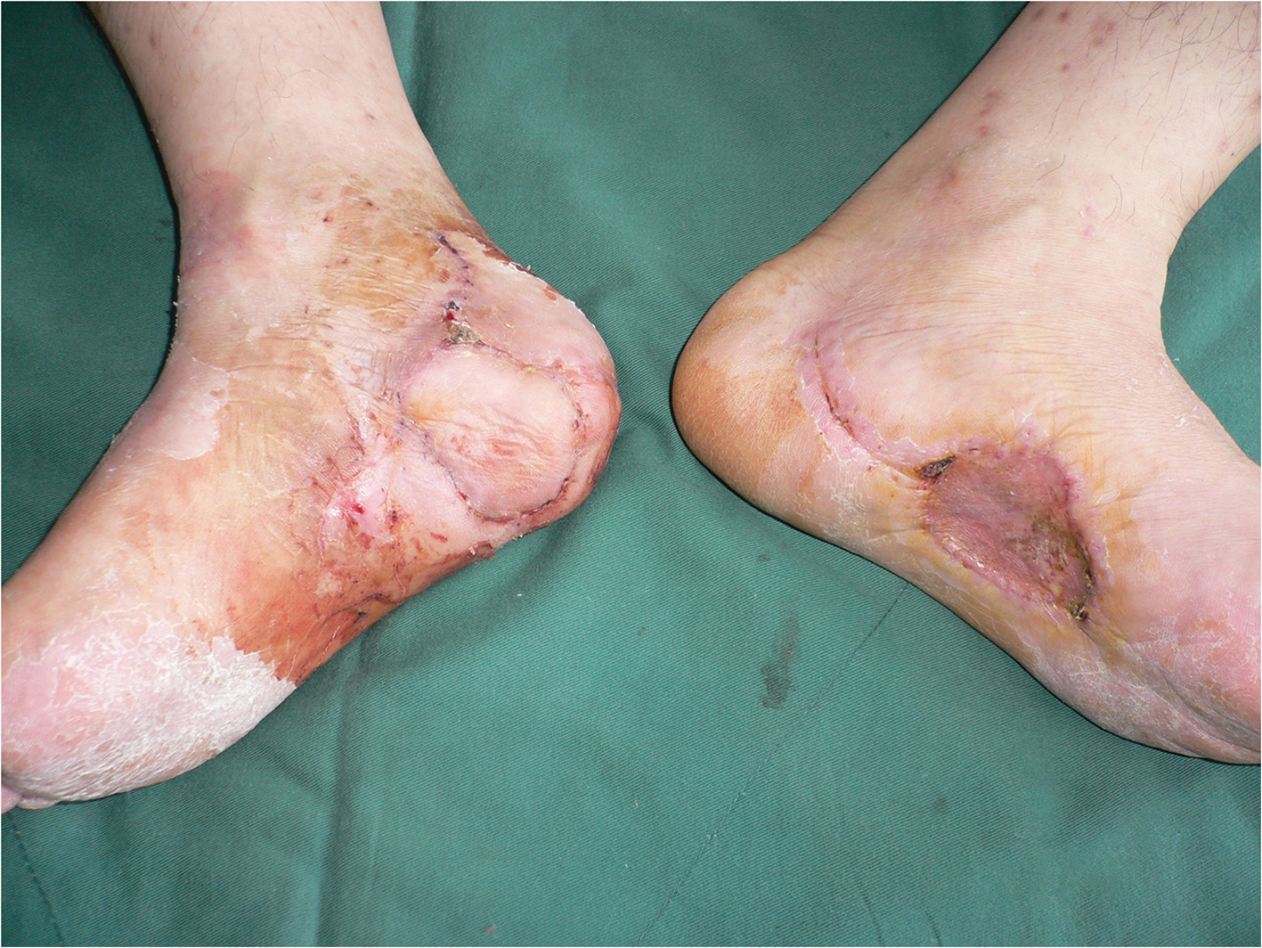
Photograph of the graft 21 days after surgery showing complete flap survival.

So as not to sacrifice an important artery in the traumatized foot of our patient, inset of the flap into the defect was followed by end-to-side anastomosis of its medial plantar artery to the recipient posterior tibial artery. The venous anastomosis was carried out end-to-end. One artery and two venae comitans were anastomosed. An epineurial window was meticulously made on the medial surface of the tibial nerve to ensure nerve-sprouting from sensory nerve fascicles. The cutaneous nerve fascicles of the flap were then anastomosed end-to-side with the recipient tibial nerve. When the tourniquet was freed, vascularization of the flap was noted (Figure 5).The donor and recipient sites healed without any complications during 3 weeks of follow-up (Figure 6). Two months after surgery, the patient was able to wear normal footwear and had a normal gait. At his 3-month follow-up examination, the patient subjectively reported an increase in sensation at the recipient site compared to immediately after surgery. Static two-point discrimination was 20mm compared to 18mm of the contralateral normal side at the 6-month follow-up visit. At 30 months after surgery, no ulceration in the flap due to poor contact with the shoe had occurred.

## Discussion

The glabrous epidermis and dermis of the heel are much thicker than skin in other regions of body, and the strong fibrous septa binding plantar fascia to the periosteum of the calcaneum create fat loculations that produce a shock-absorbing system. This special structural and functional characteristic of the skin anatomical system is crucial for the weight-bearing region of the foot in the heel. The reconstruction of a full-thickness defect in this area still represents a challenge for a plastic surgeon. A variety of options have been used to reconstruct the defects, including the reverse sural flap [2,6], the lateral supramalleolar flap [7], the lateral calcaneal flap [8], the instep flap [4,9] and different free flaps [10]. The aim in performing heel reconstruction is to provide durable coverage with a normal appearance and to make the sole regain the capability of withstanding the entire body weight and have sensation to prevent pressure sores [11]. It depends on the specialized soft-tissue coverage in this region, which should provide durable, sensitive, stable components similar to those of the defect of the original tissue. Therefore, an ideal donor site should be with similar anatomical structure to cover the soft-tissue defect, meanwhile it is a non-weight-bearing area of the sole. The instep flap makes successful heel reconstruction possible. The instep region of the foot is a source of durable plantar skin from a non-weight-bearing donor site and its use avoids exposing the donor site to weight-bearing stresses. Although other pedicled flaps can also provide coverage of a heel defect, they usually involve transfer of non-glabrous skin, which is often thick, insensitive and not as durable as glabrous plantar skin. The skin from the instep region of the foot can be used to cover heel defects as a transposition flap [9], an island fasciocutaneous flap [4] and a free flap [12]. For those cases where the transposition flap and island fasciocutaneous flap cannot be used due to vascular injuries or because extension of the defect is large, spreading to the ipsilateral instep area, the free instep flap from the contralateral foot can achieve the goal.

The construction of instep flaps is based on the medial plantar vessels. The medial plantar artery is the smaller terminal branch of the posterior tibial artery. It passes distally along the medial side of the foot, with the medial plantar nerve being lateral to it. At its origin, it lies deep into the abductor hallucis and then runs distally into the intermuscular space between it and the flexor digitorum brevis, giving muscular branches to both, and terminates by anastomosing with a branch from the first plantar metatarsal artery to the medial side of the great toe. The cutaneous supply to the instep area is provided by one to three cutaneous perforators from the medial plantar artery on each side of the abductor hallucis muscle. The first and second perforators are found to have the larger diameters. The skin territory of a free instep flap based on the first and second perforators that can be harvested extends from the metatarsal heads to the heel, with maximal flap dimensions of 12cm in length and 7cm in width. Mourougayan [11] reported that the instep flap does not receive any significant myocutaneous blood supply. The flap [12] can thus be isolated purely on its neurovascular bundle without a cutaneous or muscle transposition base. It is this fact that broadens the instep flap’s potential for use as a free flap.

Traditionally, end-to-end anastomosis of the artery of the flap to the recipient artery necessitates transection of the recipient artery. This inevitably results in permanent vascular impairment of the already traumatized foot. However, end-to-side anastomosis of the artery has been found to cause no damage to the recipient artery, which is very important for patients with a seriously injured foot. In order to avoid congestion of the flap and post-operative complications, we used two accompanying veins of the artery for anastomosis. This secured the venous return of the flap, and no flap congestion occurred.

In 1979, Shanahan and Gingrass [9] described the sensory flap based on the medial plantar artery and incorporating the medial plantar nerve for the coverage of heel defects, which could provide protective sensitivity to the weight-bearing area to reduce subsequent injury risk [13]. In this procedure, the free instep flap is centered over the first metatarsal bone and can be harvested as a sensate flap by retaining cutaneous nerve fascicles within the flap and proximal interfascicular dissection of the medial plantar nerve [12,14]. Wan *et al*. [13] examined the retention of innervations relative to the contralateral heel and instep. The amount of sensation was documented for cold temperature, light pressure, static two-point discrimination and dynamic two-point discrimination. Interestingly, he found that the sensory acuity of the damaged heel became better than normal after reconstruction. Traditionally, end-to-end coaptation of sensory fascicles of the flap to the donor nerve inevitably results in permanent sensory impairment of the skin area originally innervated by the donor nerve. Lykoudis *et al.*[15] reported that end-to-side nerve neurorrhaphy of the sensory fascicles of the free instep flap avoided sensory compromise of the forefoot and toes and that sensory sprouting was enough for flap reinnervation. This theory is consistent with our result in the present case. Though the question whether it is necessary to use sensate flaps has no clear answer, end-to-side anastomosis of the nerve in our patient proved advantageous because it obviated the delay in nerve regeneration and neurotization.

## Conclusion

The free instep flap is significantly better than the cross-foot flap and is more acceptable to patients because it never requires prolonged hospitalization and immobilization in awkward positions. The major disadvantage of the free instep flap is sacrifice of the medial plantar artery and sometimes the tibialis posterior vascular system. Fortunately, the medial plantar artery is not the dominant artery of the foot and is expendable. Moreover, surgeons frequently encounter a discrepancy in the diameter of microvascular anastomosis in the proximal foot with relatively smaller vessels, and this technique also requires a team specially trained in microvascular surgery.

## Consent

Written informed consent was obtained from the patient for publication of this case report and any accompanying images. A copy of the written consent is available for review by the Editor-in-Chief of this journal.

## Competing interests

The authors declare that they have no competing interests.

## Authors’ contributions

LL conceived of and designed the study, participated in the operation and drafted the manuscript. X(x)C performed the operation and collected the data. YZ wrote the manuscript, participated in the operation and helped to collect the data. X(c)C revised the manuscript critically. All authors read and approved the final manuscript.
